# AcSDKP Regulates Cell Proliferation through the PI3KCA/Akt Signaling Pathway

**DOI:** 10.1371/journal.pone.0079321

**Published:** 2013-11-07

**Authors:** Ping Hu, Bin Li, Wenhua Zhang, Yijian Li, Guang Li, Xinnong Jiang, Joanna Wdzieczak-Bakala, Jianmiao Liu

**Affiliations:** 1 Sino-France Laboratory for Drug Screening, Key Laboratory of Molecular Biophysics of Ministry of Education, College of Life Science and Technology, Huazhong University of Science and Technology, Wuhan, Hubei, China; 2 Institut de Chimie des Substances Naturelles, CNRS UPR2301, Gif sur Yvette, France; Sun Yat-sen University Medical School, China

## Abstract

The natural tetrapeptide acetyl-N-Ser-Asp-Lys-Pro (AcSDKP) is generated from the N-terminus of thymosin-β4 through enzymatic cleavage by prolyl oligopeptidase (POP). AcSDKP regulation of proliferation of different cells is implicated in hematopoiesis and angiogenesis. This tetrapeptide present in almost all cells was recently detected at elevated concentrations in neoplastic diseases. However, previously reported *in vitro* and *in vivo* studies indicate that AcSDKP does not contribute to the pathogenesis of cancers. Here we show that exogenous AcSDKP exerts no effect on the proliferation of actively dividing malignant cells. Using S17092, a specific POP inhibitor (POPi), to suppress the biosynthesis of AcSDKP in U87-MG glioblastoma cells characterized by high intracellular levels of this peptide, we found that all tested doses of POPi resulted in an equally effective depletion of AcSDKP, which was not correlated with the dose-dependent decreases in the proliferation rate of treated cells. Interestingly, addition of exogenous AcSDKP markedly reversed the reduction in the proliferation of U87-MG cells treated with the highest dose of POPi, and this effect was associated with activation of the phosphatidylinositol-3 kinase (PI3K)/Akt pathway. However, extracellular-regulated protein kinase (ERK) activation was unaltered by S17092 and AcSDKP co-treatment. Knockdown of individual PI3K catalytic subunits revealed that p110α and p110β contributed differently to AcSDKP regulation of U87-MG cell proliferation. Disruption of p110α expression by small interfering RNA (siRNA) abrogated AcSDKP-stimulated Akt phosphorylation, whereas knockdown of p110β expression exhibited no such effect. Our findings indicate for the first time that the PI3KCA/Akt pathway mediates AcSDKP regulation of cell proliferation and suggest a role for this ubiquitous intracellular peptide in cell survival.

## Introduction

The natural tetrapeptide AcSDKP is released in organisms from its metabolic precursor thymosin β4 by the prolyl oligopeptidase (POP) [1]. It was initially described as a physiological inhibitor of primitive hematopoietic cell proliferation [2]. This peptide also inhibits the growth of cardiac fibroblast and mesangial cells [3-5]. Additional evidence implicated AcSDKP in the promotion of angiogenesis, stimulating endothelial cell proliferation [6-8]. Our previous studies revealed elevated levels of endogenous AcSDKP in neoplastic diseases, including hematologic malignancies and solid neoplasms [9,10]. Recently, a correlation between AcSDKP expression and POP activity was shown in different types of malignant tumors [11]. These findings are in line with the overexpression of thymosin β4 in a large variety of solid neoplasms [12,13]. However, continuous *in vivo* administration of AcSDKP to animals bearing grafted solid tumors had no effect on its progression and development [10]. Moreover, it was also reported that AcSDKP was inactive on leukemic cells [14-16]. While the role that AcSDKP plays in the control of cell growth remains somewhat controversial, the molecular mechanism through which AcSDKP affects cell proliferation also remains largely unknown.

The activated PI3K/Akt/mammalian target of rapamycin (mTOR) signaling pathway provides major survival signals to normal and many cancer cells [17]. Activated Akt modulates the function of numerous substrates involved in the regulation of many cellular processes, including cell survival, cell cycle progression, and cellular growth. Class I PI3Ks in mammals comprise three distinct catalytic subunits (p110α, p110β, and p110δ) according to their structure and interaction with p85 and p55 regulatory subunits [18]. The catalytic subunits p110α and p110β are ubiquitous and may control cell division. p110α acts primarily downstream of receptor tyrosine kinase (RTK) and is found to be amplified and mutated in a wide range of solid tumors [19]. The relative importance of p110β in RTK signaling is not entirely clear, and recent studies suggest that this isoform acts mainly downstream of G protein-coupled receptors (GPCRs) [20].

In the current study, we used PTEN-deficient U87-MG glioblastoma cell line that is characterized by high intracellular concentration of AcSDKP, and investigated the mechanism of action of AcSDKP. We examined the effect of AcSDKP on the proliferation of U87-MG cells both in the absence and presence of a POP inhibitor (POPi). S17092, a specific and bioavailable POPi, has been shown to penetrate human cells and achieve a full inhibition of endogenous proline endopeptidase [21]. We found that treatment with AcSDKP alone, as expected, did not lead to apparent changes in the proliferation rate of cells, which is consistent with previously reported results [14-16] and suggests no direct implication of this peptide in malignant cell proliferation. However, our results revealed the ability of exogenous AcSDKP to rescue cell proliferation impairment in cells treated with high doses of POPi that completely suppressed the intracellular AcSDKP. Importantly, AcSDKP abolished POPi-induced inhibition of cell proliferation in parallel with increased Akt phosphorylation. Our findings provide new insights into the mechanism of action of AcSDKP in the control of cell survival.

## Materials and Methods

### Antibodies and Reagents

Antibodies for Western blot analysis, including p110α, p110β, β-actin, phospho-Akt (Ser^473^), phospho-ERK1/2 (Thr^202^/Tyr^204^), phospho-S6 (Ser^235/236^) and total-Akt, ERK1/2, S6 antibodies as well as goat anti-rabbit horseradish peroxidase (HRP)-linked and goat anti-mouse horseradish peroxidase (HRP)-linked secondary antibodies were from Cell Signaling Technology (Beverly, MA, USA). p53 antibody was from Santa Cruz Biotechnologies (Santa Cruz, CA, USA). Sterile samples of synthetic AcSDKP (MW 487, purity > 95% as determined by reverse phase high-performance liquid chromatography) were provided by Genepep (Saint-Jean de Védas, France). Institut de Recherche Servier (Courbevoie, France) has provided us the POP inhibitor, S17092. A stock solution of 100 mg/ml S17092 was prepared in dimethyl sulfoxide (DMSO) and then diluted in culture medium to obtain its final concentration before use. 0.1% DMSO has been used as the vehicle control for all the experiments. Wortmannin was purchased from Merck4Biosciences (Darmstadt, Germany).

### Cell culture and siRNA transfection

All cancer cell lines were obtained from American Type Culture Collection (Rockville, MD) and were cultured according to the supplier’s instructions. Briefly, human leukemia K562 and colorectal carcinoma HCT116 cells were grown in RPMI 1640 containing 10% FCS and 1% glutamine. Human breast carcinomas cell lines (MCF7, MDA-MB231 and MDA-MB435), A549 human lung carcinoma, U87-MG human glioblastoma and B16F10 mouse melanoma cells were grown in Dulbecco minimal essential medium (DMEM) containing 4.5 g/L glucose supplemented with 10% FCS and 1% glutamine. All cell lines were maintained at 37°C in a humidified atmosphere containing 5% CO_2_. Fetal bovine serum, culture medium, and other solutions used for cell culture were from Invitrogen (Shanghai, China).

Cells were plated at a density of 6×10^5^ cells in 60 mm diameter plates and then transfected with siRNA against p110α, p110β, or negative control (Santa Cruz, CA, USA) using Lipofectamine 2000 (Invitrogen, Shanghai, China) according to manufacturer’s instructions. Protein was isolated 96 h after transfection and the level of silencing determined by Western Blot analysis. Transfected cells were incubated in serum-free media overnight and treated with POPi and AcSDKP for the indicated periods of time.

### AcSDKP measurement

AcSDKP concentration was measured using a highly specific competitive enzyme immunoassay (EIA) with acetylcholinoesterase conjugate as a tracer (SPIbio, Massy, France), as previously described[6]. Briefly, cells were collected every day for five days and immediately supplemented with lisinopril (Sigma, Saint-Quentin Fallavier, France) at 10^-6^ M final concentration to prevent AcSDKP degradation. AcSDKP was quantified in the methanol extracts. The protein pellet was discarded and the collected supernatants were evaporated to dryness under vacuum (Speed Vac Concentrator, Savant, France). The extracts were then taken up in 0.5 ml of EIA buffer and used to determine the concentration of AcSDKP according to the manufacturer’s protocol. The results are expressed in picomoles per 1×10^6^cells.

### Cell proliferation assay

[^3^H] thymidine incorporation was assessed to determine the effects of AcSDKP on DNA synthesis. Cells were seeded onto 24-well plates at a density of 1×10^5^ cells per well and allow to adhere for 24 h. Cells were incubated in serum-free DMEM for 24 h and then treated with either S17092 alone or combined with AcSDKP (10^-13^ to 10^-5^M) for indicated periods of time. Cells were then labeled with DMEM medium containing 1µCi/ml [^3^H] thymidine (Amersham, Buckinghamshire, UK) for the last 4 h. Each well was washed once with 1 ml ice-cold PBS. Cells were fixed in 10% trichloroacetic acid (TCA) and then solubilized in 0.3M NaOH. The radioactivity corresponding to [^3^H] thymidine incorporation into the DNA was measured using a Beckman scintillation counter. 

### Western blot analysis

Cells were plated at a density of 1×10^5^ cells in 35 mm diameter plates. After 24 h, cells were incubated in serum-free media overnight and then treated with AcSDKP or other chemical reagents for indicated periods of time. Cell lysates from cultures were sonicated and the protein concentration was evaluated using the Bradford reagent (Bio-Rad Laboratories LTD, Hertfordshire, UK). Equal amounts of protein (20 μg) from each sample were separated by 12% SDS-PAGE and transferred to polyvinylidene fluoride membranes (Millipore, Bedford, MA). The membranes were blocked in blocking buffer (5% nonfat dry milk in TBS and 0.1% Tween 20) for 2h and then incubated overnight at 4°C with the indicated primary antibodies: p110α, p110β, β-actin, p-Akt, p-ERK1/2, p-S6 and total Akt, ERK1/2, S6. Antibody recognition was detected with goat anti-rabbit or anti-mouse horseradish peroxidase (HRP)-linked secondary antibodies (1:20000; Cell Signaling Technology). Immunoblots were revealed by the enhanced chemiluminescence reagents (Pierce, USA) and visualized on the X-ray film. The density of immunoreactive bands was measured using NIH image software and all bands were normalized to percentages of control values.

### Statistical analyses

Data are presented as mean ± SEM from at least 3 independent experiments. Statistical analysis of data was performed using the Student's t-test. A value of p<0.05 was considered significant.

## Results

### AcSDKP expression in different cell lines and its suppression by POPi

**Figure 1:**
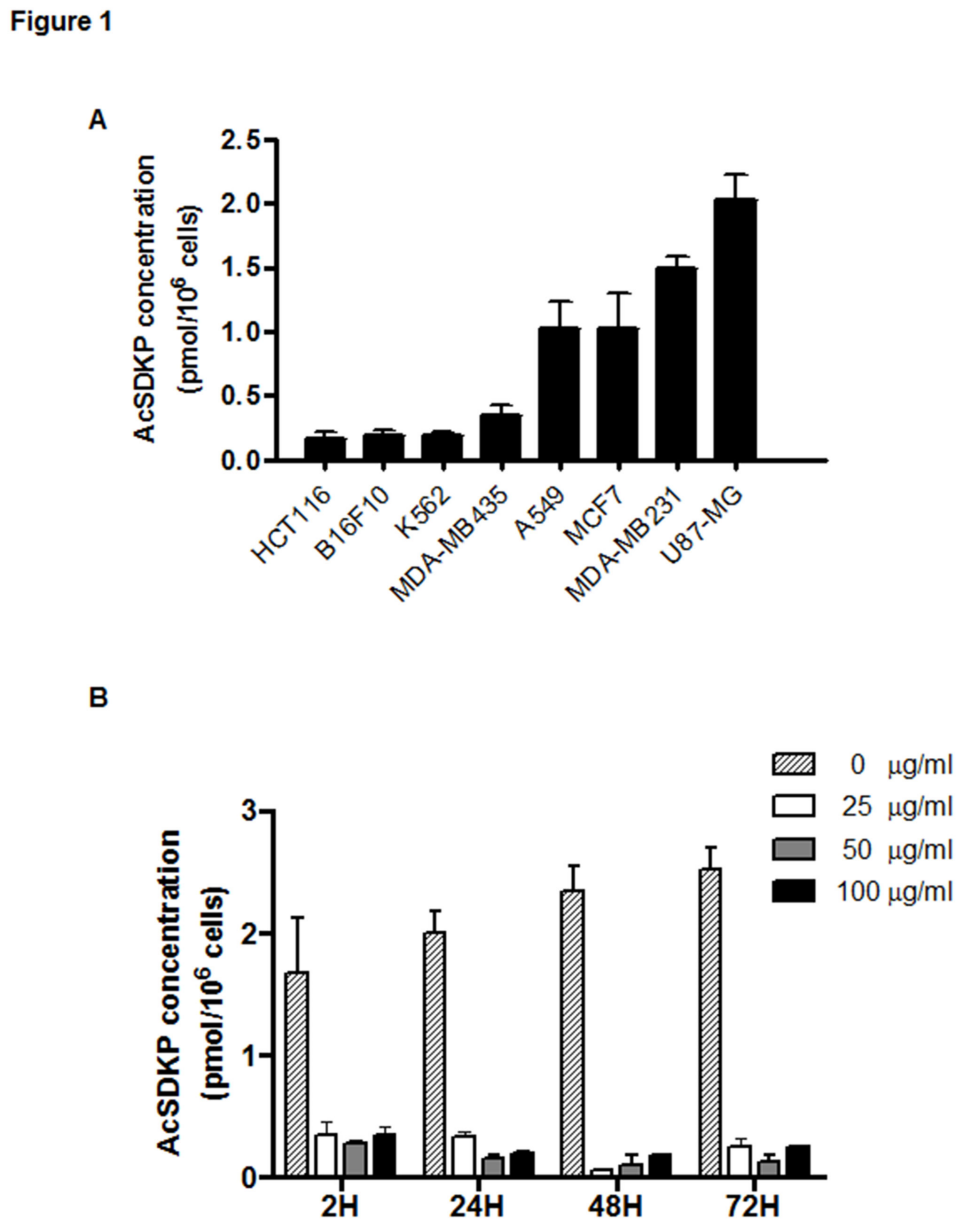
Intracellular concentration of AcSDKP. The level of endogenous AcSDKP peptide was measured daily in cells grown in 25-cm^2^ flasks in the absence or presence of POP inhibitor (POPi). Following trypsin detachment, the cells were counted, washed with PBS, sonicated, and AcSDKP was extracted with methanol according to the procedure described in “Materials and Methods”. (**A**) The AcSDKP expression in different cell lines: HCT116: human colon cancer cell; B16F10: mouse melanoma cell; K562: human leukemia cell; MCF7, MDA-MB231 and MDA-MB435: human breast cancer cell; A549: human lung cancer cell; U87-MG: human glioblastoma cell. (**B**) Kinetics of down-regulation by POPi of AcSDKP in U87-MG cells. Cells were treated by S17092 compound, an inhibitor of AcSDKP biosynthesis, at different concentrations from 2 to 72 h. The results represent the mean ± SEM from three experiments.

We first examined AcSDKP expression in a large panel of cancer cell lineages. As shown in Figure 1A, the endogenous level of AcSDKP in human U87-MG glioblastoma cells (2.024 ± 0.199 pmol/10^6^ cells) was significantly higher than in other detected cancer cells. To determine whether the observed high level of AcSDKP in malignant cells interferes with proliferation, we blocked the intracellular production of this peptide with the compound S17092, a potent POPi. The selected four cell lines, K562, HCT116, U87-MG and HuVEC, which exhibited different levels of AcSDKP, were treated with S17092 at different concentrations (from 5 to 100 µg/ml). The AcSDKP synthesis was quickly (2 h) inhibited to the similar degree by S17092 at various concentrations tested (Figure S1). AcSDKP content in U87-MG cells was then measured at different time intervals after treatment with S17092. As shown in Figure 1B, the inhibitory effect of S17092 in U87-MG cells was observed at 2 hours of treatment and remained constant for up to 72 hours.

### Exogenous AcSDKP reverses POPi-induced inhibition of proliferation of U87-MG cells

**Figure 2:**
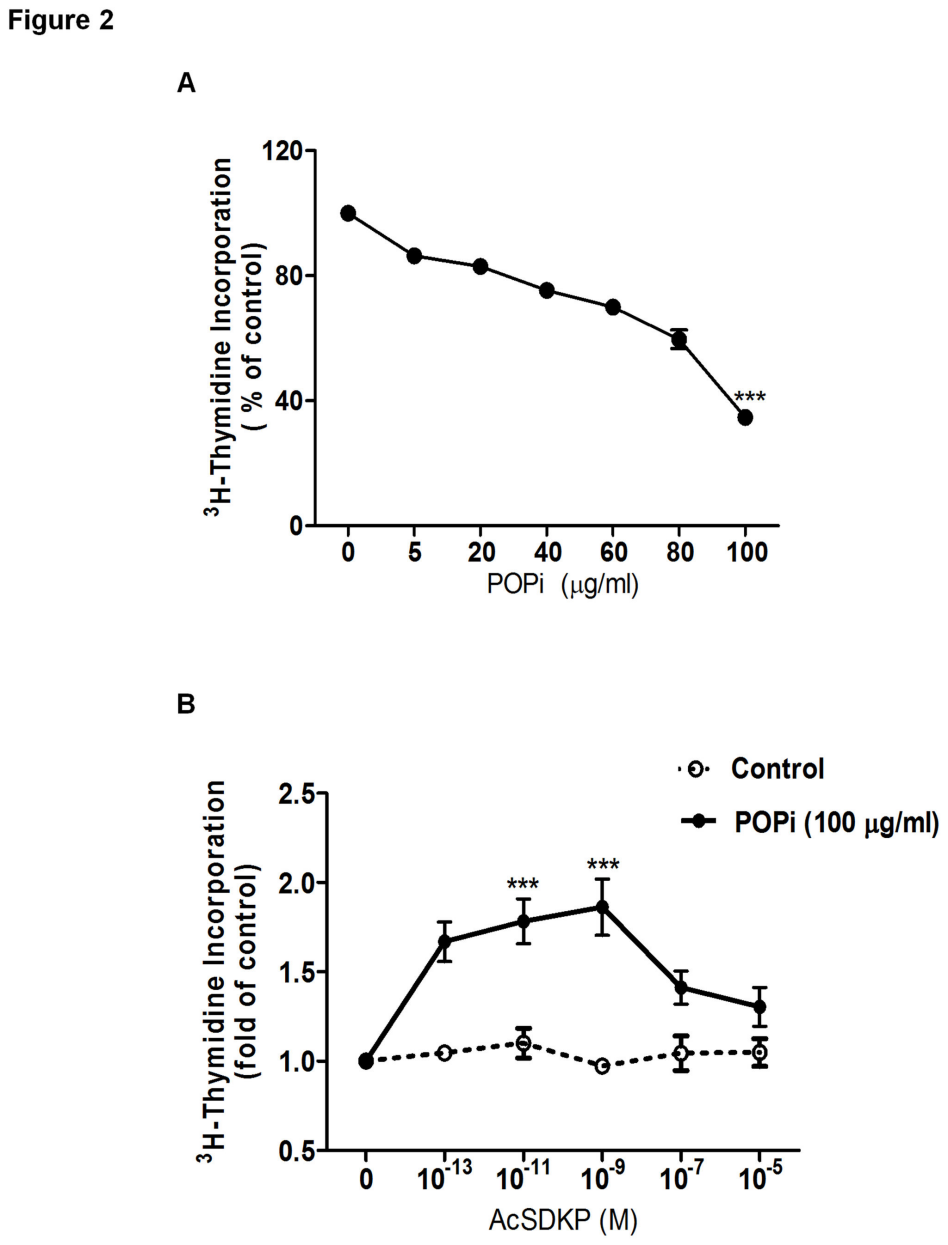
Effect of AcSDKP on proliferation of U87-MG cells in the absence or presence of S17092. (**A**) Effect of S17092 on the proliferation rate of U87-MG cells. DNA synthesis was measured by [^3^H]-thymidine incorporation assay as described in “Materials and Methods”. Cells were treated with S17092 at different concentrations for 24 h. (**B**) Cells were pretreated with 100 μg/ml of S17092 for 2 h and then combined with AcSDKP at the indicated concentrations for 24 h. The data represent least-squares mean ± SEM. Values that differ significantly from untreated cells are indicated by asterisks (***p<0.001).

Next, we examined whether suppression of AcSDKP by POPi can interfere with cell proliferation using the [^3^H] thymidine incorporation assay. As shown in Figure 2A, progressive reductions in DNA synthesis were detected following S17092 treatment in U87-MG cells, and a dose-dependent effect was observed. Moreover, S17092 displayed a similar inhibitory effect on the proliferation of other cancer cells, including MCF7 and HCT116 cells (Figure S2A and C). Notably, the observed dose-dependent anti-proliferative effect of S17092 did not correlate with the decreased level of AcSDKP (Figure 1B), which was equally suppressed at all the tested doses of POPi. These results imply the existence of potential unknown off-targets of POPi that could be responsible for the observed inhibitory effect on cell proliferation. Nonetheless, blocking endogenous AcSDKP synthesis could be employed to investigate the function of exogenous AcSDKP.

As AcSDKP alone showed no effect on DNA synthesis in U87-MG, MCF7 and HCT116 cells (Figure 2B, S2B and S2D), we then investigated the effect of exogenous AcSDKP on cell proliferation in the presence of S17092. Interestingly, addition of exogenous AcSDKP to cells that was firstly treated with the highest concentration of S17092 (100 μg/ml) revealed a cell type-selective restoring effect on the proliferative state of treated cells. We observed that POPi-induced reduction of DNA synthesis could be rescued by exogenous AcSDKP in U87-MG cells, but not in MCF7 and HCT116 cells (Figure 2B, S2B and S2D). Moreover, AcSDKP restored DNA synthesis in a biphasic and dose-dependent manner, with its maximum effect observed at 10^-9^ M. Of note, the observed effect of AcSDKP was progressively diminished at doses higher than 10^-9^ M, while no apparent stimulatory effect was seen at the dose of 10^-7^ M.

### AcSDKP regulates U87-MG cell proliferation via the PI3K/Akt pathway

**Figure 3:**
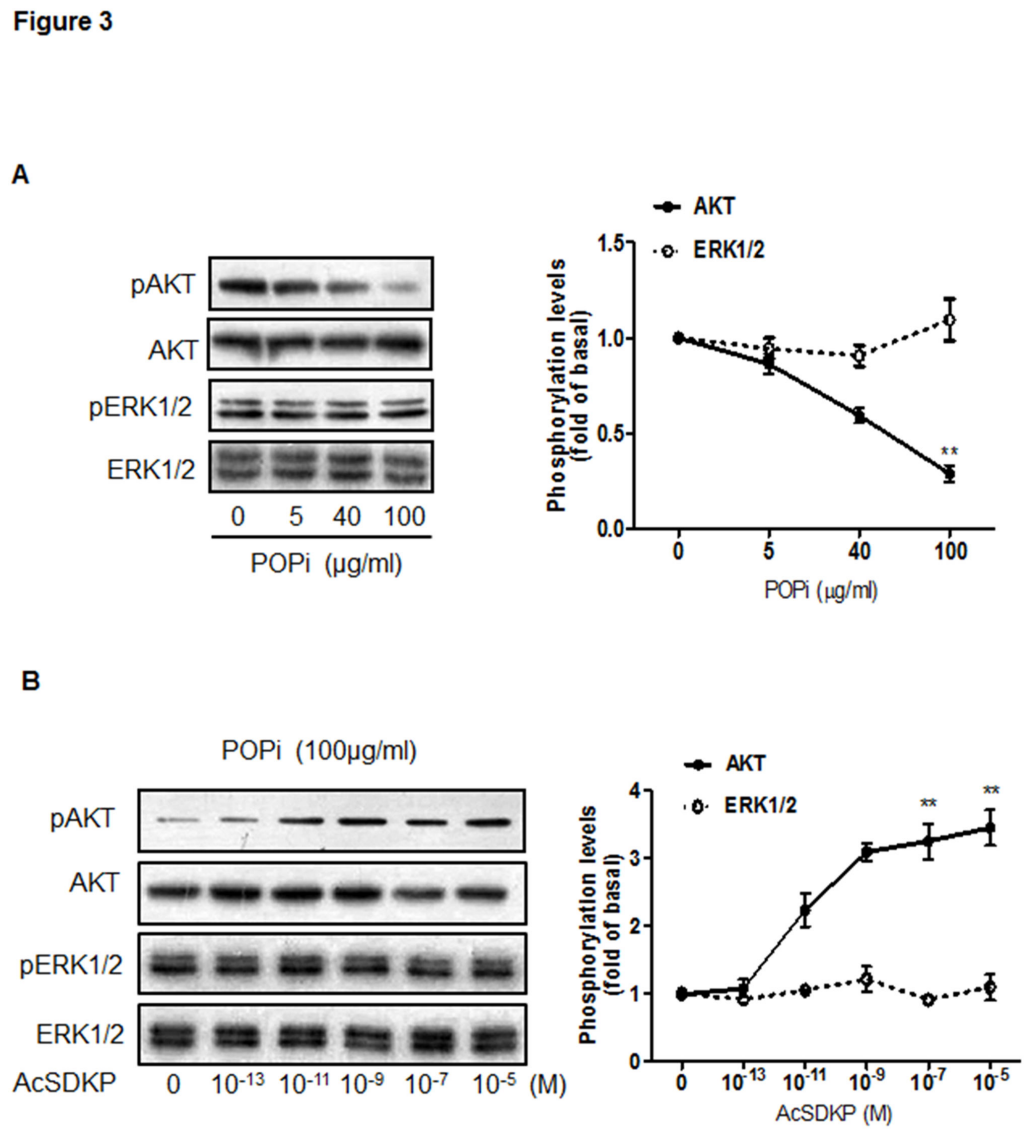
AcSDKP restored the proliferation of U87-MG cells through Akt activation. (**A**) Effect of S17092 on the phosphorylation of Akt (Ser^473^) and ERK1/2 in U87-MG cells. Cells were incubated with different concentrations of S17092 for 2 h and analyzed by Western blot with antibodies against p-Akt and p-ERK1/2 proteins. (**B**) Effect of AcSDKP on the phosphorylation of Akt and ERK1/2 in S17092-treated U87-MG cells. Cells were pretreated with 100 μg/ml of S17092 for 2 h and then combined with AcSDKP at the indicated concentrations for 2 h. The graph represents the relative level of Akt and ERK1/2 phosphorylation compared with the control group. Similar results were obtained from three independent experiments. The ratio of band intensities was calculated after normalizing the phospho-protein signals with the total protein signals. The calculations were done using NIH image software.

**Figure 4:**
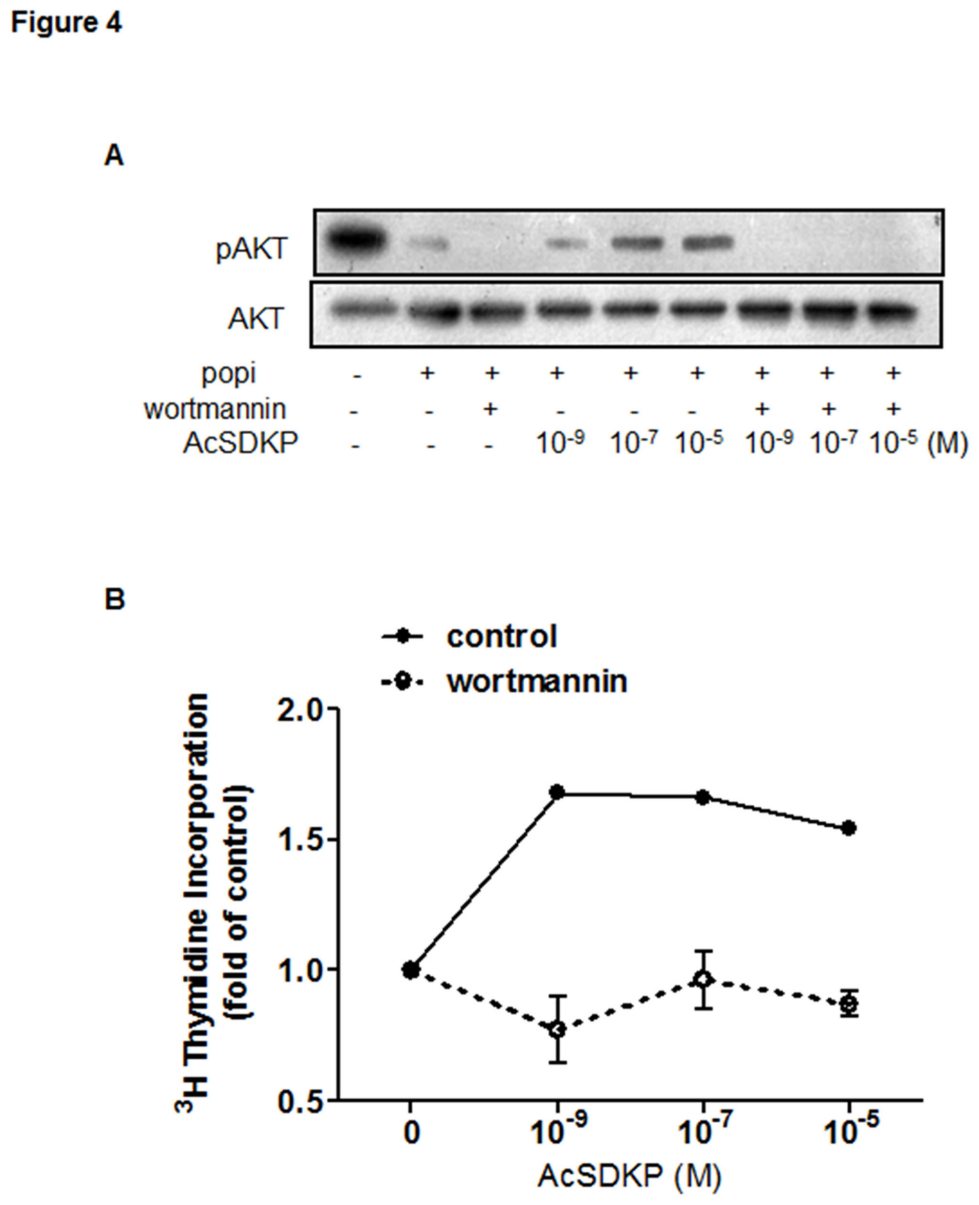
Effect of wortmannin, a PI3K inhibitor, on AcSDKP-induced Akt phosphorylation and U87-MG proliferation. Cells were pretreated with 100 μg/ml S17092 for 90 min and then with 10 μM wortmannin for additional 30 min before addition of different concentrations of AcSDKP in the presence of 100 μg/ml S17092. (**A**) Effect of wortmannin after co-treatment for 2 h with POPi and AcSDKP was analyzed by Western blot for p-Akt and Akt proteins. (**B**) Cell proliferation was determined after co-treatment for 24 h with POPi and AcSDKP by [^3^H]-thymidine incorporation assays performed in triplicate. Representative results are shown from three independent experiments.

Given the ability of AcSDKP to significantly reverse POPi-induced impairment of proliferation in U87-MG cells, we further investigated the signaling pathway(s) through which this peptide exerts its effect. As Mitogen-Activated Protein Kinases (MAPK) and PI3K/Akt pathways have been shown to critically regulate cell proliferation and survival, we examined Akt and ERK1/2 phosphorylation levels following treatment of U87-MG cells with POPi (100 μg/ml). In comparison to untreated cells, significantly reduced Akt phosphorylation was detected after 2 hours of POPi treatment, while ERK1/2 phosphorylation remained unchanged (Figure 3A). As shown in Figure 3B, addition of exogenous AcSDKP to POPi-pretreated U87-MG cells resulted in robust increases in Akt phosphorylation in a dose-dependent fashion. By contrast, AcSDKP showed no effect on ERK1/2 phosphorylation. Additionally, Akt or ERK1/2 phosphorylation remained unchanged when cells were treated only with AcSDKP (Figure S3). To confirm the participation of PI3K in the signaling pathway, we used a specific inhibitor of PI3K activation (wortmannin, 100 nM), which blunted AcSDKP-induced Akt phosphorylation (Figure 4A). The presence of wortmannin also led to a loss of AcSDKP’s effect on U87-MG cell proliferation as examined by ^3^H incorporation assay (Figure 4B). These results suggest that observed effect of AcSDKP on DNA synthesis may be dependent in part upon its restoration of PI3K/Akt signaling.

### p110α but not p110β mediates AcSDKP regulation of Akt phosphorylation and cell proliferation

**Figure 5:**
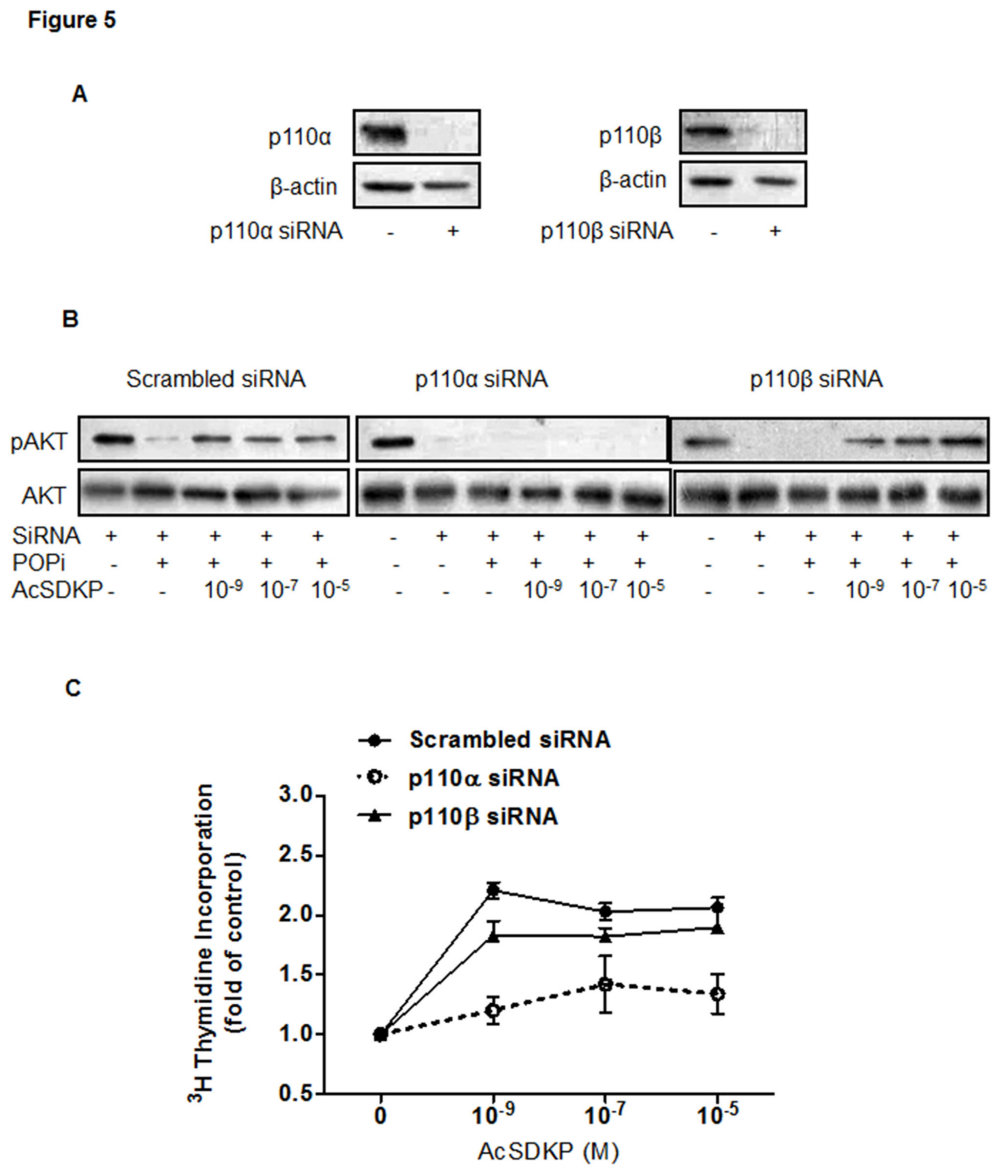
AcSDKP activated Akt phosphorylation via p110α but not p110β. (**A**) RNA interference effectively reduced PI3K p110α and p110β protein expression in U87-MG cells. At 4 days post transfection, activation of Akt was evaluated by Western blot. β-actin was used as the loading control. (**B**). Extracts from U87-MG cells transfected with p110 siRNA or scrambled RNA control were analyzed by Western blot for p-Akt and Akt proteins after co-treatment with POPi and AcSDKP as indicated. Representative results are shown from three independent experiments. (**C**) The effect of AcSDKP on proliferation of cells transfected with p110 siRNA or scrambled RNA control. Cell proliferation was determined by [^3^H]-thymidine incorporation assays performed in triplicate.

To gain insights into the molecular mechanism that mediates the observed effect of AcSDKP on cell proliferation, we focused on the Akt signal transduction pathway in U87-MG cells. Given that the catalytic p110α and p110β isoforms of PI3K are ubiquitously expressed, we investigated which subunit of PI3K was involved. Using specific siRNAs directed against p110α or p110β, the expression of the two targeted proteins was effectively suppressed after 96 h transfection as compared with scramble siRNA control (Figure 5A). Remarkably, AcSDKP-induced increase in Akt phosphorylation was significantly blocked in p110α knockdown cells but not in p110β knockdown cells (Figure 5B). Cell proliferation assays also corroborated the involvement of p110α but not p110β in the effect of AcSDKP upon U87-MG cell proliferation (Figure 5C). These results indicate that p110α plays an important role in AcSDKP-induced Akt phosphorylation and cell proliferation.

## Discussion

AcSDKP regulates several critical cellular functions, including cell cycle progression, cell migration, invasion and angiogenesis. AcSDKP has been shown to inhibit PDGF-BB or fetal calf serum (FCS)-induced DNA synthesis by increasing the stability of p53 protein, inhibit the proliferation of isolated cardiac fibroblasts, but significantly stimulate endothelial cell proliferation in a concentration-dependent manner. AcSDKP is present in all studied alive cells and has been shown to be highly expressed in sprouting endothelial cells. Its high level in cancer cells and tissues was also reported [10,11]. However, the role of AcSDKP overexpression in the malignancies has not been elucidated. It is possible that the high levels of AcSDKP in malignant cells could contribute to the symptomatology and/or aetiology of the cancer. Nevertheless, all the previously reported findings have yet to provide evidence for the direct implication of AcSDKP in malignant development. In the current study, we report a significantly higher concentration of AcSDKP in U87-MG glioblastoma cells relative to other tested cancer cells. This prompted us to have yet another attempt to elucidate the molecular mechanism by which AcSDKP may control cell proliferation.

Our data demonstrate that (1) AcSDKP alone in the normal cell growth conditions had no effect on the proliferation of U87-MG cells; (2) POPi suppressed almost completely endogenous AcSDKP in the treated U87-MG cells at the used doses; (3) POPi inhibited U87-MG cell proliferation in a dose-dependent manner; (4) exogenous AcSDKP markedly restored U87-MG cell growth when impaired by POPi; (5) the PI3KCA/Akt pathway was involved in AcSDKP regulation of cell proliferation, while ERK appeared to be uninvolved; (6) p110α, but not p110β, was implicated in mediating AcSDKP-stimulated Akt phosphorylation. The observed biphasic and dose-dependent stimulatory effect of AcSDKP was similar to its activity profile previously reported for the rat cardiac fibroblast [3] and human endothelial cell [6]. Indeed, the activity of this tetrapeptide occurs at low concentrations and disappears at higher concentrations. Although we cannot currently explain this biphasic effect, such a bell-shaped curve of AcSDKP activity might suggest that this peptide activates multiple receptors or enzymes, with each of them leading to either stimulation or inhibition. 

To investigate the implication of AcSDKP in cell proliferation and the underlying molecular mechanism, we have exploited the property of an efficient inhibitor of POP which allows the modulation of the intracellular level of the tetrapeptide. POP is a serine endoprotease implicated in the hydrolysis of many bioactive proline-containing peptides such as angiotensins, neurotensin, arginine-vasopressin, substance P and thryotropin releasing hormone [22]. It is the main enzyme responsible for *in vivo* generation of AcSDKP [1]. The proliferation of U87-MG cells impaired by the highest dose of POPi was markedly recovered by the supplementation of culture medium with exogenous AcSDKP. This effect was mediated by the PI3K/Akt pathway activation. Consequently, the observed inhibition of U87-MG cell proliferation induced by POPi was associated with down-regulation of PI3K/Akt pathway. It has been previously shown that cytoplasmic POP can control inositol-1,4,5-triphosphate (IP_3_) synthesis by negatively regulating multiple inositol polyphosphate polyphosphatase (MInsPP), which generates IP_3_ from IP_5/6_ [23,24]. Thus, POP inhibition increases the cellular pool of inositol by elevating the formation of IP_3_, which is known as a second messenger for releasing intracellular calcium, from higher order inositol phosphates. Inositol phosphates including IP_4_, IP_5_ and IP_7_ have been reported to antagonize Akt signaling. The lifetime of IP3 is very short (less than 30 sec) before being transformed into IP2 and then IP1. Therefore, we performed the experiments to measure the level of IP1 in U87-MG cell. As expected, POPi enhanced the accumulation of IP1 in a dose-dependent manner. However, no effect of AcSDKP on the IP1 level in presence of POPi was observed (Figure S4). The second messenger IP3 causes the release of Ca^2+^ from intracellular stores and activates the protein kinase C (PKC). It is not surprising that AcSDKP contributes to the activation of Akt (PKB) in our experimental conditions without any influence on the activity of PKC. Inhibition of AcSDKP production occurred at a 4-fold lower concentration of POPi than that needed for inhibition of Akt phosphorylation, and this suggests that down-regulation of AcSDKP may partly block PI3K/Akt signaling and there may exist unknown off-targets of POPi that are likely responsible for the observed inhibitory effect on Akt activation..

Treatment of U87-MG cells with POPi inhibited Akt activation. The extent of this inhibition was reduced by co-treatment with AcSDKP through PI3K/Akt pathway. It is well known that the PI3K/Akt pathway is relevant for diverse fundamental cellular functions, such as cell proliferation, survival and mobility, and is frequently aberrantly activated in cancer cells [17,25]. Glioblastoma multiformes frequently show mutant forms or loss of the phosphatase and tensin homologue (PTEN), which is correlated with high levels of phosphorylated Akt [26,27]. The signaling pathways downstream of PI3K were elucidated through the use of pan-specific inhibitor, such as wortmannin. It irreversibly blocks p110α via a covalent interaction with a critical lysine residue Lys^802^ [28]. In this study, we demonstrated the implication of AcSDKP in the U87 cell proliferation impaired by POPi and its effect on activation of the PI3K/Akt pathway. The use of small interfering RNA (siRNA) against p110α allowed us to affirm the role of p110α in mediating AcSDKP’s effect on POPi-elicited reduction of cell proliferation. In fact, the activity of AcSDKP on Akt phosphorylation and cell proliferation was partly blocked by depletion of p110α, but not p110β. It is well known that p110α functions primarily downstream of RTK and p110β is activated downstream of GPCRs. It is tempting to speculate that the involvement of AcSDKP in the control of cell growth is mediated by a specific receptor(s) which can subsequently activate p110α. 

Our current findings suggest the implication of AcSDKP in the control of cell proliferation with the potential to activate the PI3KCA/Akt pathway. It would be interesting to elucidate if mTOR and p53, downstream effectors of Akt, were affected by POPi treatment. We found that phosphorylation of S6, one of the two specific substrates of mTOR, and p53 expression did not change after POPi treatment in the absence or presence of AcSDKP (Figure S5). However, it is also conceivable that AcSDKP could activate other proteins, making them potential targets for the regulation of cell proliferation. There is an emerging concept that all intracellular signaling cascades do not follow linear paths to their destinations, but rather form a network of signaling circuits to regulate cell functions [29]. Further investigations are needed to define the relative contributions of Akt or other potential AcSDKP-interacting proteins in the proliferation and growth of U87-MG cells regulated by AcSDKP.

In conclusion, our results highlight that the ability of AcSDKP to interfere under particular cell conditions with U87-MG cell growth. We demonstrate for the first time that AcSDKP influences cell proliferation via the PI3K/Akt signaling pathway. Understanding and exploration of key regulators of cell proliferation or cell survival may lead to potential targets for cancer therapy.

## Supporting Information

Figure S1 **Effect of POPi on intracellular AcSDKP concentration.** The endogenous level of peptide was measured daily in cells grown in 25cm^2^ flasks in the absence or presence of POP inhibitor (POPi). Following trypsin detachment, the cells were counted, washed with PBS, sonicated, and AcSDKP was extracted with methanol according to the procedure described in “Materials and Methods”. Effect of S17092 on AcSDKP biosynthesis was examined in HuVEC, U87-MG, HCT116 and K562 cells at different indicated concentrations for 2 hours. The results are the mean ± SEM of 3 experiments.
(TIF)

Figure S2 **No effect of AcSDKP on proliferation of HCT116 and MCF7 cells in the absence or presence of S17092.** (**A**, **C**) Effect of S17092 on the proliferation rate of HCT116 and MCF7 cells. DNA synthesis was measured by [^3^H]-thymidine incorporation assay as described in “Materials and Methods”. Cells were treated with different concentrations of S17092 for 24h. (**B**, **D**) Cells were pretreated with 100 μg/ml of S17092 for 2h and then combined with indicated concentrations of AcSDKP for 24h in comparison with the controls. The data represents least-squares mean ± SEM. Values that differ from untreated cells are indicated by asterisks (***p<0.001).
(TIF)

Figure S3 **No effect of exogenous AcSDKP on the Akt and ERK1/2 phosphorylation in U87-MG cells.** Cells were incubated with different concentrations of AcSDKP for 2h and analyzed by Western blot with antibodies against p-Akt and p-ERK1/2 proteins. Similar results were obtained from three independent experiments.
(TIF)

Figure S4 **No effect of AcSDKP on IP1 Accumulation in U87-MG cells.** IP1 accumulation was determined using the IP-One kit (Cisbio, Bioassays, Bagnols sur-Cèze, France). Cells were seeded onto 96-well plates at a density of 1×10^4^ cells per well. After 24 h, cells were incubated in serum-free media overnight. After treatment, the fluorescence (HTRF) components were then added: the europium cryptate-labeled anti-IP1 antibody, and the d2-labeled IP1 analogue were both diluted in lysis buffer. The energy transfer signals were measured 50s after excitation at 320 at both 620 and 665 nm, respectively, using the 2103 EnVision^TM^ Multiabel Plate Readers (Perkin Elmer,Waltham,MA,USA). The results are calculated from the 665nm/620nm ration, which is inversely proportional to the concentration of IP1 in the cells, was then transformed into the accumulated IP1 value using a calibration curve prepared on the same plate. (**A**) Effect of S17092 on IP1 Accumulation in U87-MG cells. Cells were treated with different concentrations of S17092 for 2 h. (**B**) Effect of AcSDKP on IP1 Accumulation in the presence of POPi. Cells were pretreated with 100 μg/ml of S17092 for 2h and then combined with indicated concentrations of AcSDKP for 2h in comparison with the controls. The data represents least-squares mean ± SEM. Values that differ from untreated cells are indicated by asterisks (***p<0.001).
(TIF)

Figure S5 **No effect of AcSDKP on p53 and S6 phosphorylation in U87-MG cells.** Cells were pretreated with 100 μg/ml of S17092 for 2h or then combined with different indicated concentrations of AcSDKP for 2h. It was analyzed by Western blot with antibodies against p53 and p-S6 proteins. Similar results were obtained from three independent experiments.
(TIF)
