# Supplementary figures and images for: AcSDKP Regulates Cell Proliferation through the PI3KCA/Akt Signaling Pathway
Source: PLoS One. 2013 Nov 7;8(11):e79321. doi: 10.1371/journal.pone.0079321 (PMC3820705; doi:10.1371/journal.pone.0079321)

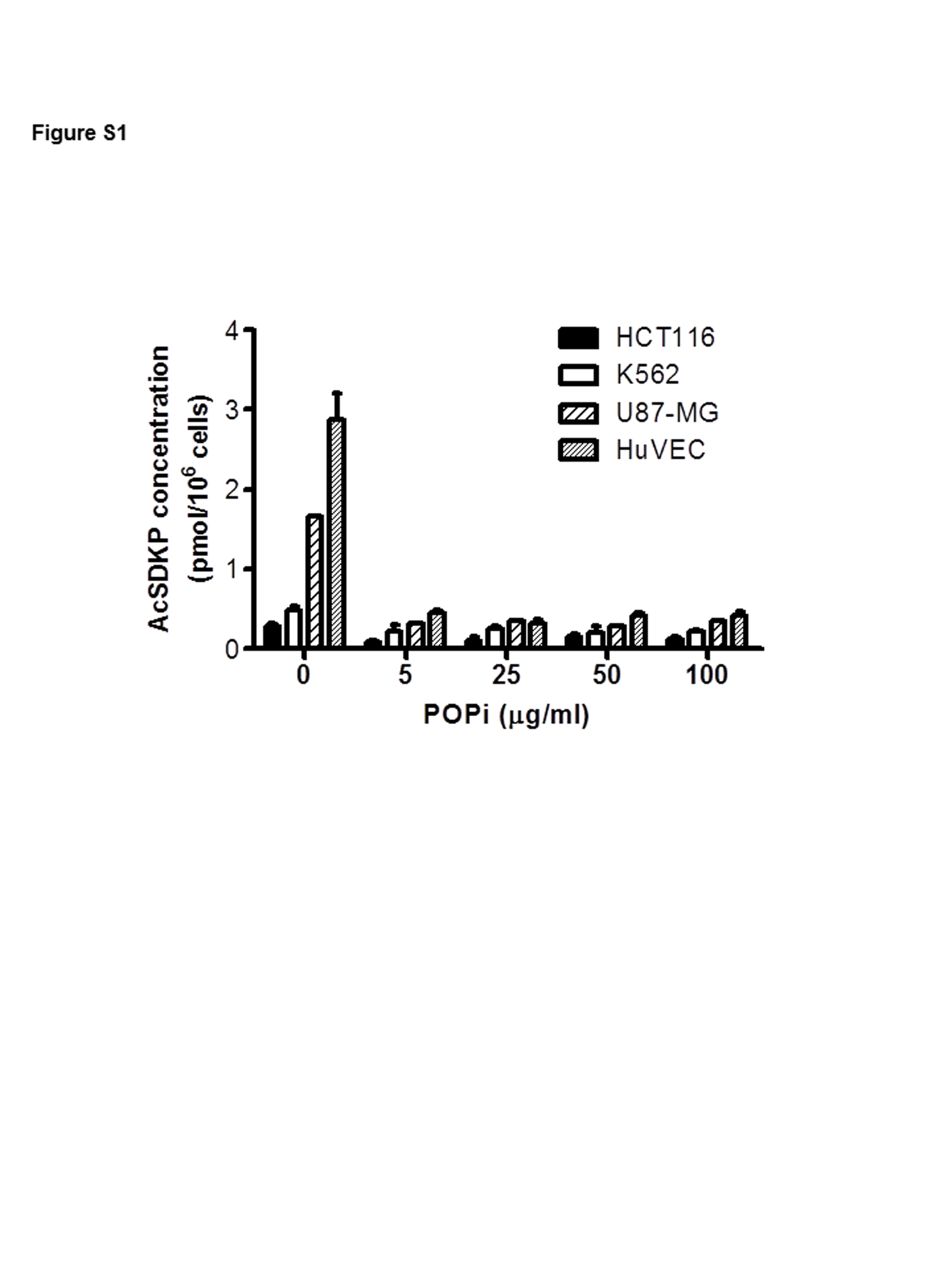

Supplement: Figure S1 — Effect of POPi on intracellular AcSDKP concentration. The endogenous level of peptide was measured daily in cells grown in 25cm2 flasks in the absence or presence of POP inhibitor (POPi). Following trypsin detachment, the cells were counted, washed with PBS, sonicated, and AcSDKP was extracted with methanol according to the procedure described in “Materials and Methods”. Effect of S17092 on AcSDKP biosynthesis was examined in HuVEC, U87-MG, HCT116 and K562 cells at different indicated concentrations for 2 hours. The results are the mean ± SEM of 3 experiments. (TIF) [file pone.0079321.s001.tif]

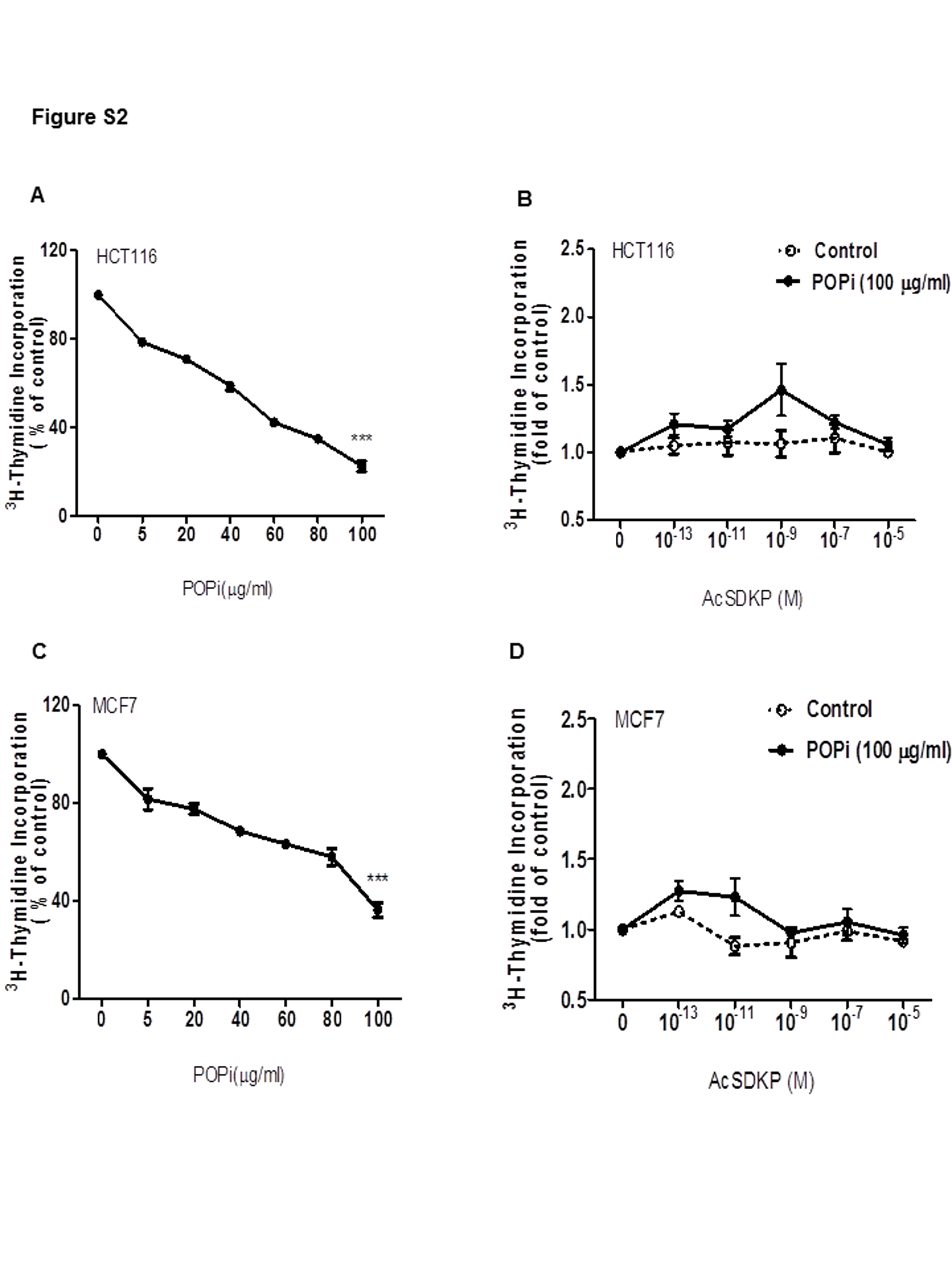

Supplement: Figure S2 — No effect of AcSDKP on proliferation of HCT116 and MCF7 cells in the absence or presence of S17092. (A, C) Effect of S17092 on the proliferation rate of HCT116 and MCF7 cells. DNA synthesis was measured by [3H]-thymidine incorporation assay as described in “Materials and Methods”. Cells were treated with different concentrations of S17092 for 24h. (B, D) Cells were pretreated with 100 μg/ml of S17092 for 2h and then combined with indicated concentrations of AcSDKP for 24h in comparison with the controls. The data represents least-squares mean ± SEM. Values that differ from untreated cells are indicated by asterisks (***p<0.001). (TIF) [file pone.0079321.s002.tif]

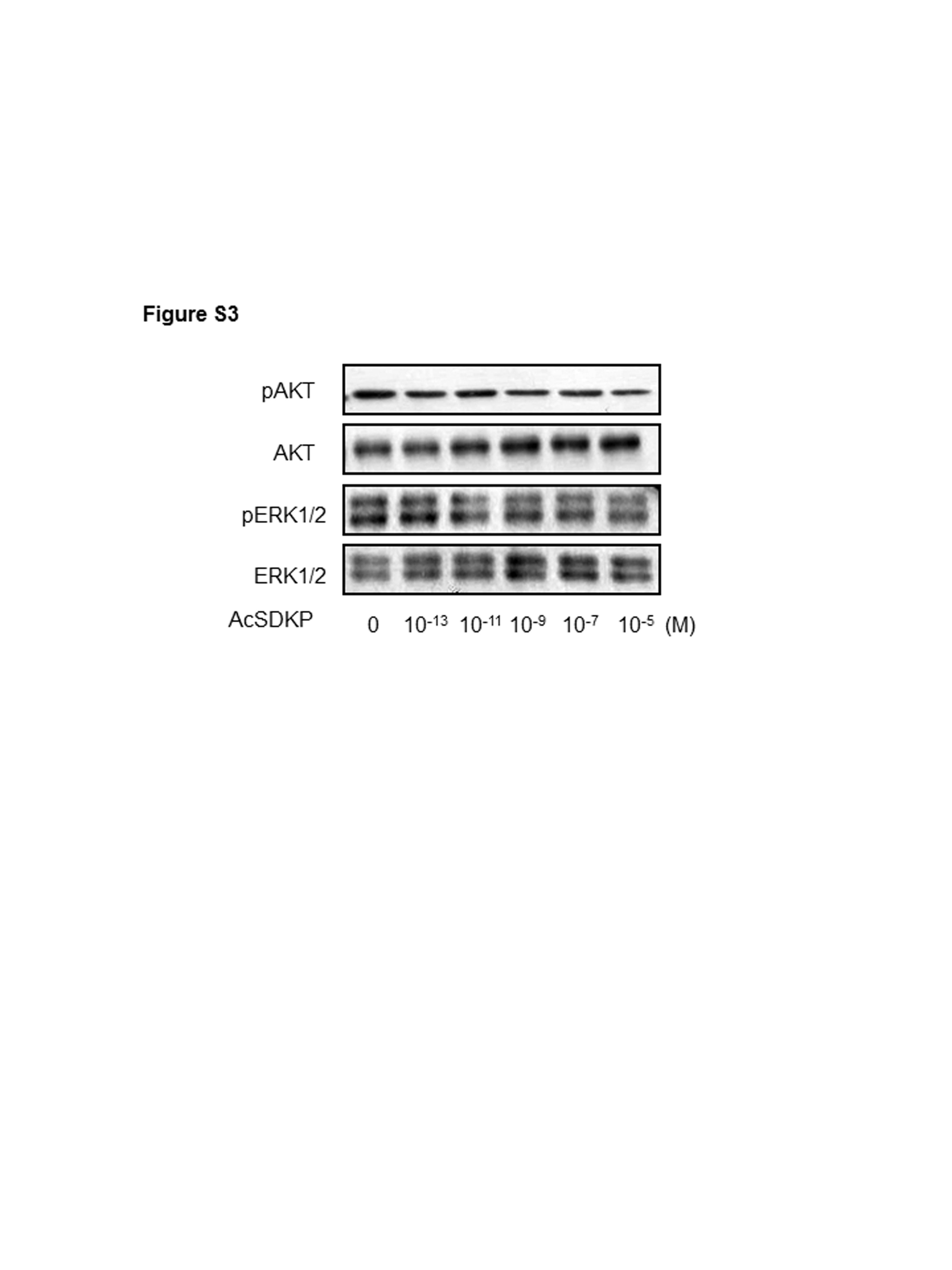

Supplement: Figure S3 — No effect of exogenous AcSDKP on the Akt and ERK1/2 phosphorylation in U87-MG cells. Cells were incubated with different concentrations of AcSDKP for 2h and analyzed by Western blot with antibodies against p-Akt and p-ERK1/2 proteins. Similar results were obtained from three independent experiments. (TIF) [file pone.0079321.s003.tif]

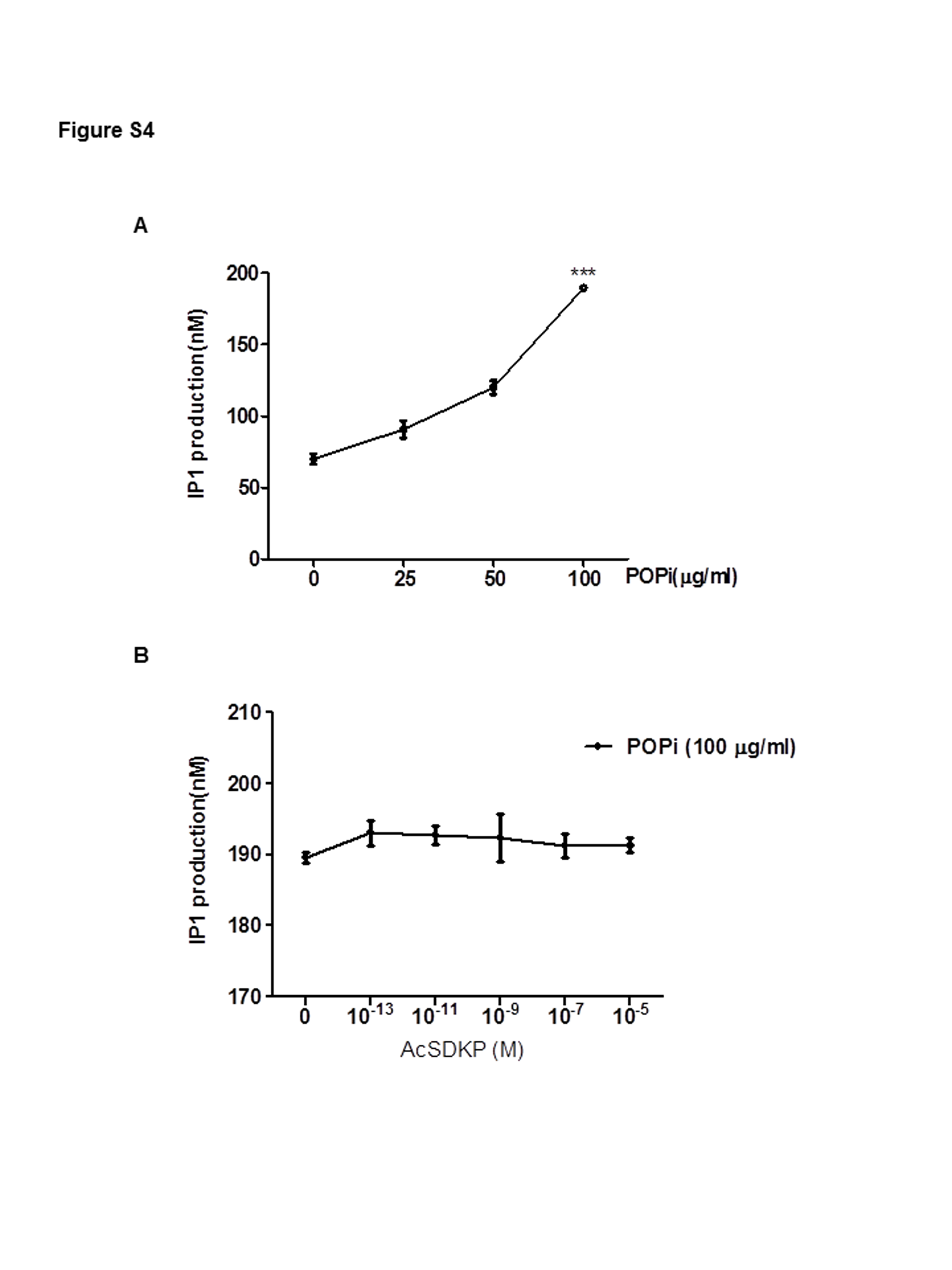

Supplement: Figure S4 — No effect of AcSDKP on IP1 Accumulation in U87-MG cells. IP1 accumulation was determined using the IP-One kit (Cisbio, Bioassays, Bagnols sur-Cèze, France). Cells were seeded onto 96-well plates at a density of 1×10^4^ cells per well. After 24 h, cells were incubated in serum-free media overnight. After treatment, the fluorescence (HTRF) components were then added: the europium cryptate-labeled anti-IP1 antibody, and the d2-labeled IP1 analogue were both diluted in lysis buffer. The energy transfer signals were measured 50s after excitation at 320 at both 620 and 665 nm, respectively, using the 2103 EnVisionTM Multiabel Plate Readers (Perkin Elmer,Waltham,MA,USA). The results are calculated from the 665nm/620nm ration, which is inversely proportional to the concentration of IP1 in the cells, was then transformed into the accumulated IP1 value using a calibration curve prepared on the same plate. (A) Effect of S17092 on IP1 Accumulation in U87-MG cells. Cells were treated with different concentrations of S17092 for 2 h. (B) Effect of AcSDKP on IP1 Accumulation in the presence of POPi. Cells were pretreated with 100 μg/ml of S17092 for 2h and then combined with indicated concentrations of AcSDKP for 2h in comparison with the controls. The data represents least-squares mean ± SEM. Values that differ from untreated cells are indicated by asterisks (***p<0.001). (TIF) [file pone.0079321.s004.tif]

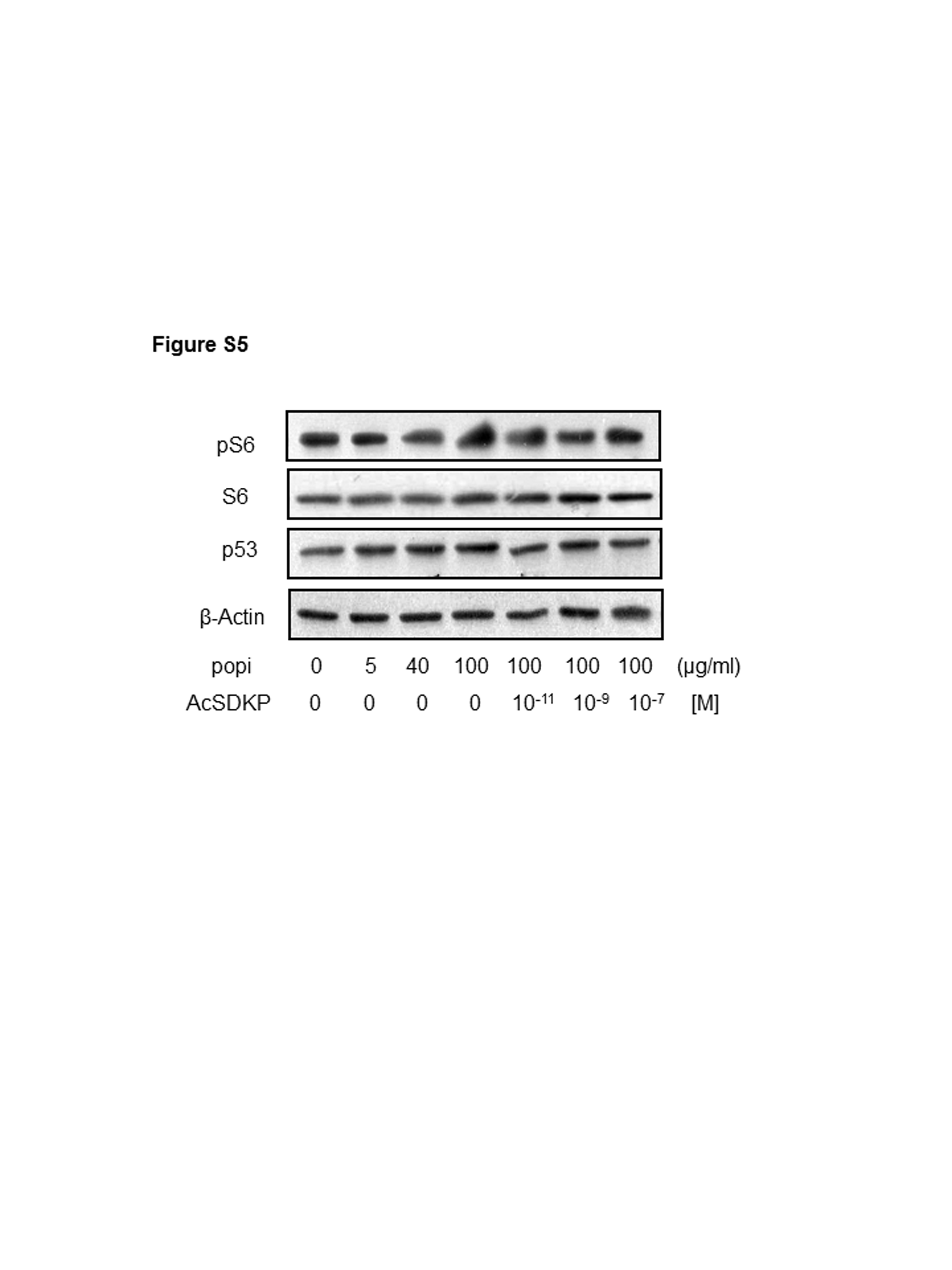

Supplement: Figure S5 — No effect of AcSDKP on p53 and S6 phosphorylation in U87-MG cells. Cells were pretreated with 100 μg/ml of S17092 for 2h or then combined with different indicated concentrations of AcSDKP for 2h. It was analyzed by Western blot with antibodies against p53 and p-S6 proteins. Similar results were obtained from three independent experiments. (TIF) [file pone.0079321.s005.tif]
